# A novel inert crystal delivery medium for serial femtosecond crystallography

**DOI:** 10.1107/S2052252515009811

**Published:** 2015-06-30

**Authors:** Chelsie E. Conrad, Shibom Basu, Daniel James, Dingjie Wang, Alexander Schaffer, Shatabdi Roy-Chowdhury, Nadia A. Zatsepin, Andrew Aquila, Jesse Coe, Cornelius Gati, Mark S. Hunter, Jason E. Koglin, Christopher Kupitz, Garrett Nelson, Ganesh Subramanian, Thomas A. White, Yun Zhao, James Zook, Sébastien Boutet, Vadim Cherezov, John C. H. Spence, Raimund Fromme, Uwe Weierstall, Petra Fromme

**Affiliations:** a Department of Chemistry and Biochemistry, Arizona State University, PO Box 871604, Tempe, AZ 85287-1604, USA; b Center for Applied Structural Discovery, The Biodesign Institute, PO Box 875001, Tempe, AZ 85287-5001, USA; c Department of Physics, Arizona State University, PO Box 871604, Tempe, AZ 85287-1504, USA; d SLAC National Accelerator Laboratory, 2575 Sand Hill Road, Menlo Park, CA 94025, USA; e Center for Free-Electron Laser Science, Deutsches Elektronen-Synchrotron DESY, Notkestrasse 85, 22607 Hamburg, Germany; f Department of Physics, University of Wisconsin-Milwaukee, 1900 East Kenwood Boulevard, Milwaukee, WI 53211, USA; g Bridge Institute, Department of Chemistry, University of Southern California, 3430 S. Vermont Avenue, Los Angeles, CA 90089, USA

**Keywords:** serial femtosecond crystallography, viscous crystal delivery, protein complexes, membrane proteins, femtosecond studies, nanocrystals, coherent X-ray diffractive imaging, free-electron laser

## Abstract

Serial femtosecond crystallography (SFX) has opened a new era in crystallo­graphy by permitting nearly damage-free, room-temperature structure determination of challenging proteins such as membrane proteins. In SFX, femtosecond X-ray free-electron laser pulses produce diffraction snapshots from nanocrystals and microcrystals delivered in a liquid jet, which leads to high protein consumption. A slow-moving stream of agarose has been developed as a new crystal delivery medium for SFX. It has low background scattering, is compatible with both soluble and membrane proteins, and can deliver the protein crystals at a wide range of temperatures down to 4°C. Using this crystal-laden agarose stream, the structure of a multi-subunit complex, phycocyanin, was solved to 2.5 Å resolution using 300 µg of microcrystals embedded into the agarose medium post-crystallization. The agarose delivery method reduces protein consumption by at least 100-fold and has the potential to be used for a diverse population of proteins, including membrane protein complexes.

## Abstract 2

Viscous sample delivery that decreases the net protein consumed in serial femtosecond crystallography is described. The agarose stream has a low background, is compatible with membrane proteins and can be used at a wide range of temperatures.

## 1. Introduction   

Serial femtosecond crystallography (SFX) is a novel structural biology technique that allows challenging protein structures to be solved from submicrometre/micrometre crystals at room temperature (Chapman *et al.*, 2011 ▸). In SFX, nanocrystals and/or microcrystals are delivered in a liquid (DePonte *et al.*, 2008 ▸) or a viscous stream (Weierstall *et al.*, 2014 ▸) into the beam path of a hard X-ray free-electron laser (XFEL). XFEL radiation is composed of femtosecond pulses typically delivered at a rate of 1–120 Hz, and diffraction patterns are obtained before the crystals are destroyed (Neutze *et al.*, 2004 ▸; Barty *et al.*, 2012 ▸). SFX currently requires large data sets because the diffraction patterns are acquired from individual randomly oriented protein crystals. Most SFX experiments thus far have been based on protein crystals delivered using a gas dynamic virtual nozzle (GDVN), where the crystals are delivered to the X-ray beam in their mother liquor (DePonte *et al.*, 2008 ▸). The gas-focused GDVN liquid jet moves at a velocity of 10–20 m s^−1^, which delivers crystals much faster than required to replenish the protein crystals between X-ray pulses at a pulse repetition rate of 120 Hz. Therefore, approximately only one out of every 10 000 crystals is probed by the X-ray pulses (Weierstall *et al.*, 2014 ▸). This type of liquid jet can consume 10–100 mg of protein for the collection of a complete data set, which is particularly problematic for membrane proteins and other proteins that can only be produced in small amounts.

Membrane proteins are an important class of proteins that are of very high relevance in biology, compromising 60% of all current drug targets (Hopkins & Groom, 2002 ▸). However, structure determination of membrane proteins lags far behind soluble protein structure determination, with less than 550 unique membrane protein structures determined so far out of over 100 000 structures currently deposited in the PDB. Membrane proteins are insoluble in water and therefore have to be extracted from the membrane in the form of protein–detergent micelles. Most membrane protein structures are obtained by either crystallization in solution in the form of protein–detergent micelles or crystallization in the lipidic environment of the lipidic cubic phase (LCP), a method for membrane protein crystallization pioneered by Landau & Rosenbusch (1996 ▸). LCP is a liquid crystalline phase that is spontaneously formed upon mixing monoacylglycerols (MAGs) and water, producing a continuous three-dimensional network of curved bilayers arranged into a cubic lattice with two networks of interconnecting continuous aqueous channels (Caffrey, 2015 ▸). The architecture of the lipid formation encourages type 1 crystal packing and has similar properties to the native cell membrane (Caffrey, 2015 ▸). Crystallization in LCP has been successful for structure determination of a wide range of membrane proteins, including microbial rhodopsins, photosynthetic complexes, β-barrels, enzymes, transporters, ion channels and especially G-protein-coupled receptors (GPCRs), a class of membrane proteins with high medicinal impact (Cherezov, 2011 ▸). A new crystal delivery system has ben developed for SFX which allows the delivery of crystals grown in LCP to the XFEL beam (Weierstall *et al.*, 2014 ▸). The high viscosity of LCP results in a much slower flow rate of the stream, thus drastically decreasing the net mass of protein needed for structure determination by SFX. LCP as a delivery medium has been successfully used to determine GPCR structures using an XFEL (Liu *et al.*, 2013 ▸; Fenalti *et al.*, 2015 ▸; Weierstall *et al.*, 2014 ▸; Zhang *et al.*, 2015 ▸). Crystallization of membrane proteins in LCP has been highly optimized, contributing to the structures of over 60 unique membrane proteins to date (Caffrey & Cherezov, 2009 ▸). However, it has been challenging to crystallize large multi-domain membrane complexes in LCP owing to the curvature associated with the lipid bilayer and the low diffusion constants of large membrane protein complexes in LCP. To date, the majority of membrane protein structures solved by X-ray crystallography have been determined from crystals of protein–detergent micelles grown in solution, which have also been successfully used for SFX experiments (Chapman *et al.*, 2011 ▸; Aquila *et al.*, 2012 ▸; Johansson *et al.*, 2012 ▸, 2013 ▸; Kupitz *et al.*, 2014 ▸). These membrane protein crystals were delivered either with the GDVN liquid injector (DePonte *et al.*, 2008 ▸), requiring large amounts of protein, the gel injector (lipidic cubic phase injector; Weierstall *et al.*, 2014 ▸) or an electrospinning injector (Sierra *et al.*, 2012 ▸), which uses less protein but uses high electric fields which could be problematic for crystal stability.

To date, all membrane protein structures delivered in LCP for SFX (Weierstall *et al.*, 2014 ▸; Liu *et al.*, 2013 ▸; Fenalti *et al.*, 2015 ▸; Zhang *et al.*, 2015 ▸) have been based on crystals that were grown in LCP. Mixing of membrane protein crystals grown in the form of a protein–detergent micelle with LCP typically leads to dissolution of the crystals, very likely caused by partitioning of the detergent, which forms the protein–detergent micelle, into the lipidic phase. This leads to depletion of the detergent in the protein–detergent micelles in the crystals, resulting in denaturation of the protein. Recently, two other viscous media, a mineral oil-based grease and petroleum jelly, have been described as alternative crystal delivery carriers (Sugahara *et al.*, 2015 ▸; Botha *et al.*, 2015 ▸). The grease mixture (Sugahara *et al.*, 2015 ▸) has been used to deliver crystals to the XFEL beam for SFX data collection of soluble model proteins at the SPring-8 Compact Free Electron Laser (SACLA XFEL), while petroleum jelly (Botha *et al.*, 2015 ▸) has been used to deliver lysozyme at the Swiss Light Source (SLS). Both of these delivery methods have so far only been demonstrated at ambient pressure and they produce significant and undesirable Debye–Scherrer rings in the region of 3.77–5 Å. No results have been presented to date that show either medium to be suitable for the delivery of multi-protein complexes or membrane proteins. Thus, it is highly desirable to develop an inert medium for the delivery of both soluble and membrane proteins to the XFEL beam at slow flow rates.

Here, we have explored and developed a new delivery medium for SFX based on agarose. Agarose is a versatile polysaccharide polymer. Extracted from seaweed, agarose dissolves in water at high temperatures (above 85°C) and forms a network of helical strands upon cooling, resulting in the formation of a gel material (Arnott *et al.*, 1974 ▸). We show here that crystals grown *via* traditional crystallization methods (*i.e.* vapour diffusion and dialysis) can be embedded into agarose post-crystallization and prior to injection. Crystal delivery in agarose can be accomplished in an expansive temperature range from 4 to 30°C, allowing crystallization conditions over a wide range of temperatures to be compatible with crystal delivery. In contrast, Mebiol, a medium recently suggested as a viscous carrier by Botha *et al.* (2015 ▸), is only viscous at temperatures above 25°C. We show here that crystals of complex membrane proteins such as the photosynthetic protein complexes photosystem I (PSI) and photosystem II (PSII) can be delivered in an agarose stream. Thus, agarose has the potential to be a general crystal delivery medium for SFX for both soluble and membrane proteins.

To test whether agarose could be used to deliver crystals of large, multi-protein complexes, phycocyanin (PC) was chosen as a model system. PC is a cyanobacterial antennae protein, part of the light-harvesting complex, which channels excitation energy to PSII, subsequently driving charge separation across the thylakoid membrane, the membrane that contains PSI and PSII. The PC complex forms a disc-like trimer in which each monomer is composed of two subunits, α and β (Schirmer *et al.*, 1985 ▸).

## 2. Materials and methods   

### 2.1. Protein purification and crystallization   

PC was isolated from *Thermosynechococcus elongatus*. Briefly, the protein was obtained by disrupting a concentrated suspension of cells using a microfluidizer at 124 MPa. The resulting suspension was further purified by ultracentrifugation at 50 000*g* for 1 h, in which large particles and aggregates were separated from the supernatant. The supernatant was then concentrated using Amicon Ultra-15 spin filters (Millipore, 100 kDa cutoff), in which most small cytosolic proteins are separated as they flow through the filters. PC was crystallized by free interface diffusion as described by Kupitz *et al.* (2014 ▸) for PSII (Saridakis & Chayen, 2003 ▸). The precipitant solution consisting of 1.0 *M* ammonium sulfate, 40 m*M* 2-(*N*-morpholino)ethanesulfonic acid (MES) pH 6.4 was added dropwise at 1 µl s^−1^ to an equal volume of protein solution (15 mg ml^−1^). Crystals of 1–5 µm in size formed after 1 d and were confirmed *via* second-order nonlinear imaging of chiral crystals (Kissick *et al.*, 2011 ▸). Prior to embedding the crystals in the agarose medium, the crystals were filtered through a 10 µm stainless-steel filter.

PSI was isolated and purified in principle as described by Fromme & Witt (1998 ▸) and Hunter & Fromme (2011 ▸) using crystallization at low ionic strength as the last purification step. The crystals were stabilized in a low ionic strength buffer that consisted of 5 m*M* MES pH 6.4, 0.02% β-dodecylmaltoside (β-DDM). PSII was isolated and purified as described by Kupitz *et al.* (2014 ▸). The concentrated protein was subjected to a series of batch crystallization steps with decreasing concentrations of precipitant, as described in Kupitz *et al.* (2014 ▸). The crystals were permitted to grow for 24 h and crystal growth was then terminated by the removal of the supernatant and the addition of buffer containing low salt (100 m*M* 1,4-piperazinediethanesulfonic acid pH 7.0, 5 m*M* CaCl_2_, 10 m*M* tocopherol, 20% PEG 2000).

### 2.2. Preparation of the agarose and embedding of crystals into the viscous medium   

After a broad screening and optimization process, a solution of 5.6%(*w*/*v*) agarose and 30% glycerol was determined to form the most stable extrusion stream. In order to obtain these conditions after mixing, 7%(*w*/*v*) ultralow-gelling-temperature agarose (Sigma–Aldrich, catalog No. A5030) was dissolved in a solution of 30% glycerol and the crystallization buffer in a 15 ml centrifuge tube and submerged in a water bath filled with boiling water for 30 min. To draw up the agarose into a 100 µl syringe (Hamilton, Model 1710), the syringe was warmed by drawing up and quickly ejecting boiling water 10–15 times (to ensure the integrity of the syringe, we avoided dipping more than the needle in solutions at temperatures higher than 80°C). The agarose was then drawn up from a 15 ml centrifugation tube that remained submerged in the water bath. For PC, the agarose was dissolved in 600 µl glycerol and 1.4 ml of a solution consisting of 15% PEG 2000, 30 m*M* MgCl_2_, 75 m*M* HEPES pH 7.0. For PSII, the agarose was dissolved in 600 µl glycerol and 1.4 ml 100 m*M* PIPES pH 7.0, 5 m*M* CaCl_2_, 16%(*w*/*w*) PEG 2000. For PSI, the agarose was dissolved in 1.4 ml 5 m*M* MES pH 6.4, 0.02% β-DDM, 600 µl 2.0 *M* sucrose. In the case of data collected at helium ambient pressure, 2 ml 5 m*M* MES pH 6.4, 0.02% β-DDM was used. 20 µl of the boiling hot agarose solution pertaining to the protein system was drawn up into a syringe. The agarose was allowed to equilibrate to room temperature for approximately 20 min before 5 µl protein crystals were mixed throughout the agarose using a syringe coupler (Cheng *et al.*, 1998 ▸); at least 40 syringe-mixing exchanges were performed or until the crystals were visually homogenously distributed in the agarose medium.

### 2.3. Data collection   

Data were collected using the CXI instrument at the Linac Coherent Light Source at SLAC (Boutet & Williams, 2010 ▸). A continuous stream of agarose with crystals embedded was extruded from a 50 µm capillary into the X-ray interaction region using the LCP injector (Weierstall *et al.*, 2014 ▸) at a flow rate of 160 nl min^−1^.

### 2.4. Data processing   

During 6 h of protein crystal screening experiments at LCLS, diffraction patterns were collected from different protein crystals (PC, PSI and PSII). PC was chosen as a model system and a complete data set was collected from PC crystals delivered in agarose medium in ∼72 min. The 513 848 detector readouts were background-corrected and the hits were filtered out using *Cheetah* (Barty *et al.*, 2014 ▸), yielding 41 100 diffraction patterns that contained 25 or more Bragg spots (an average hit rate of 8%). 14 143 patterns were indexed (*i.e.* an indexing yield of 34%) and integrated using *CrystFEL* (White *et al.*, 2012 ▸; Kirian *et al.*, 2011 ▸) with a hexagonal lattice type with unit-cell parameters *a* = *b* = 153.4, *c* = 39.6 Å (see Table 1 ▸). The merohedral space group of the crystals, *P*6_3_, exhibited an indexing ambiguity which was resolved by *ambigator*, an implementation within *CrystFEL* of an algorithm related to that described by Brehm & Diederichs (2014 ▸). We decided on a resolution cutoff at 2.5 Å based on the multiplicity and the CC* value (Karplus & Diederichs, 2012 ▸; see Table 1 ▸). The merged data set (truncated at 2.5 Å resolution) was phased by molecular replacement (MR) using *phenix.phaser* (McCoy, 2007 ▸) with PDB entry 4gy3 as the search model (after removing waters and ligands). The MR model was first refined using a segmented rigid-body protocol in which each subunit was considered as a rigid entity using *phenix.refine*. A total of ten cycles of positional, individual *B*-factor refinement, including two cycles of simulated-annealing refinement, were then performed. In this step, water molecules were added and refined using *phenix.refine* at 2.5 Å resolution. The refined structure resulted in an *R*_work_ of 18.7% and an *R*_free_ of 25.5% (see Table 1 ▸ and Supplementary Table S1). In order to demonstrate that agarose is a suitable delivery medium for SFX data collection from soluble proteins and various membrane proteins, we have also provided diffraction patterns from PSI and PSII (see Supplementary Figs. S3 and S4).

## 3. Results and discussion   

For a viscous medium to be suitable for SFX, three primary requirements must be met: the medium must maintain crystal integrity, must form a stable and continuous stream and the diffraction from the medium should produce minimal background scattering. Several viscous media were tested in order to investigate their potential as a general viscous, non-Newtonian, carrying medium, including tapioca corn starch, gelatine, silica hydrogel, polyacrylamide, polyvinyl alcohol and poly(ethylene oxide). None of these media established stable streams. Initial tests using agarose as a crystal delivery medium showed signs of dehydration in vacuum, leading to the formation of ice as detected by X-ray diffraction. To prevent freezing, we tested several potential cryoprotectants for crystal delivery in high vacuum. From those screened, we narrowed our selection to glycerol, which also increased the viscosity of the agarose stream, a welcome side effect that makes extrusion more reliable. Different concentrations of agarose and glycerol were screened to optimize the extrusion conditions and 5.6% agarose dissolved in 30% glycerol was found to be the most suitable medium because it formed a stable, continuous stream and no ice-crystal diffraction artefacts were observed (Supplementary Fig. S1).

**Figure 1:**
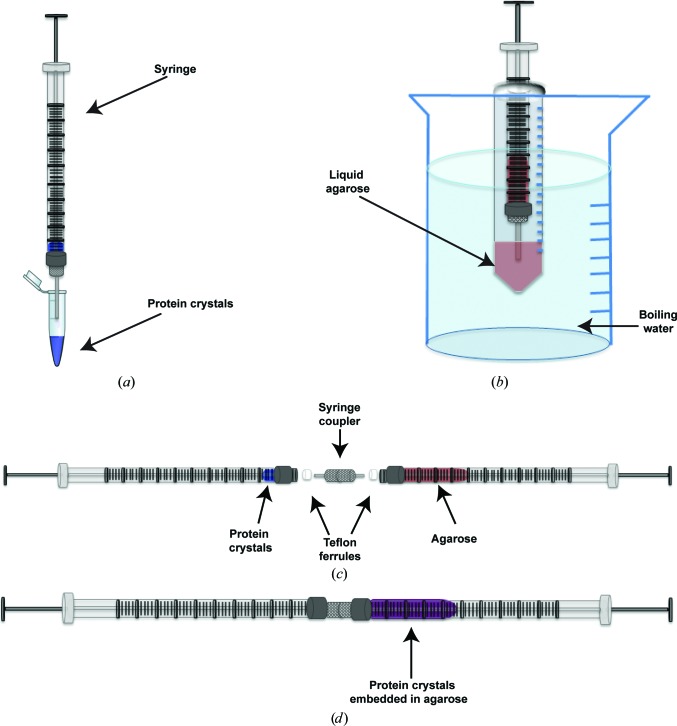
Diagram showing how the crystals are embedded into the agarose medium. (*a*) A dense pellet of crystals is drawn up into a syringe, (*b*) the agarose solution (contained in a 15 ml centrifuge tube) is submerged in boiling water until the agarose dissolves, the liquid agarose is drawn up into a warmed syringe and the agarose is allowed to gel and equilibrate to room temperature, (*c*) the protein crystals and agarose syringe are connected by a syringe coupler and (*d*) using the syringe coupler, the crystals are embedded throughout the agarose by moving the plungers back and forth.

The next challenge was to explore the best way to embed the crystals into the agarose. We explored three ways to embed the crystals into the agarose medium: (i) the growth of crystals in agarose, (ii) simple manual mixing on a glass slide (as has been used for the oil-based grease method; Sugahara *et al.*, 2015 ▸) and (iii) the use of a syringe setup (Fig. 1 ▸), which was originally developed for the crystallization of proteins in LCP (Cheng *et al.*, 1998 ▸; Caffrey & Cherezov, 2009 ▸). The growth of crystals in agarose has been described in the literature as a method to slow down crystal growth and to counteract effects such as sedimentation and convection that influence the crystallization process under gravity (Biertümpfel *et al.*, 2002 ▸). We first investigated the growth of PC crystals in agarose. Owing to the large size of PC, its diffusion constant is very low and crystal growth in agarose is very slow, leading to the formation of few nuclei. Furthermore, no nucleation occurred in agarose in the presence of 30% glycerol even at very high protein concentrations (>20 mg ml^−1^). Although PC could not be crystallized directly in the gel owing to its large size and low diffusion constant, other proteins might be suitable for crystal formation inside the agarose gel as demonstrated previously (García-Ruiz *et al.*, 2001 ▸; Lorber *et al.*, 2009 ▸).

While the growth of crystals of PC in agarose was very difficult, we succeeded in embedding pre-grown crystals into the agarose medium, which allowed a full SFX data set to be collected from PC crystals delivered in agarose (Supplementary Fig. S2). The mixing of pre-formed crystals is therefore suggested as the preferred method of crystal delivery in the agarose stream, as it does not depend on the size of the protein and allows pre-grown crystals to be delivered to the XFEL beam. However, simple mixing of crystals with agarose leads to an inhomogeneous distribution of crystals and further problems, including partial drying of crystals, the formation of crystals of salt or other precipitants and the loss of protein crystals during transfer to the injector crystal reservoir. We have therefore adapted the LCP syringe setup for embedding crystals into the agarose medium, as shown in Fig. 1 ▸. In order to stabilize the crystals, the agarose solution was prepared in the crystallization buffer corresponding to each of the proteins. The agarose was dissolved by vortex mixing the mother liquor and glycerol (for sample delivery in vacuum) with the agarose powder in a 15 ml centrifuge tube. The tube was then submerged in a water bath of boiling water for approximately 30 min. A syringe was then warmed by drawing up boiling water from the water bath all the way through the syringe and quickly ejecting it 10–15 times. With the centrifuge tube submerged in boiling water, 20 µl agarose was drawn up into the heated syringe and allowed to cool to ambient temperature. A second syringe was filled with 5 µl of the highly concentrated PC crystal suspension (ideally 10^11^ crystals per millitre) in the crystallization mother liquor (the same mother liquor as used in the agarose preparation). The desired high crystal densities can be achieved by either sedimentation or low-speed centrifugation of the crystal suspensions prior to mixing with the agarose medium (see §[Sec sec2]2 for details).

After the agarose had entered the gel phase in the syringe, the syringes containing agarose and the protein crystals were connected using a syringe coupler (Fig. 1 ▸; Cheng *et al.*, 1998 ▸). The crystals were then embedded into the agarose by alternate movement of the two plungers, whereby the solutions moved back and forth between the two syringes at least 40 times (Supplementary Fig. S2), leading to the embedding of the crystals in the agarose medium at a crystal density of 2 × 10^10^ crystals per millitre. Owing to the dilution caused by mixing of the agarose medium with the crystal suspension, the medium becomes less viscous, and the initial percentage of agarose must be increased to compensate for the dilution. Thus, the initial agarose concentration was increased to 7% agarose in order to achieve a final concentration of 5.6% after mixing with the crystals.

**Figure 2:**
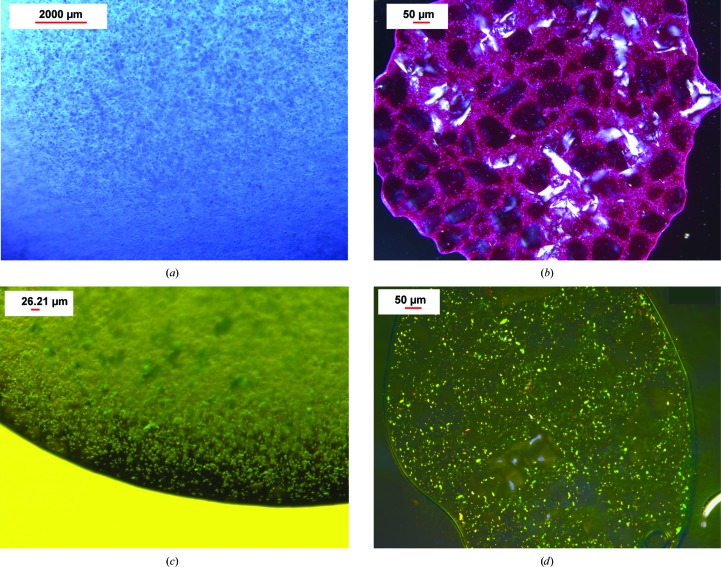
Protein crystals before and after mixing with agarose. (*a*) PC microcrystals, (*b*) PC crystals after mixing with agarose (birefringent), (*c*) PSII microcrystals (birefrigent), (*d*) PSII crystals after mixing with agarose (birefrigent).

We used the same procedure of embedding crystals of soluble model proteins such as lysozyme and the large protein–cofactor complex PC, as well as one of the largest and most complex membrane protein complexes that has been crystallized so far: PSII (a dimer of 700 kDa containing 38 protein subunits and more than 100 cofactors). Pictures of the different crystals embedded in agarose are shown in Fig. 2 ▸ and Supplementary Figs. S2 and S4. A large variety of different precipitants commonly used for crystallization are compatible with the agarose medium, including high salt concentrations as well as polyethylene glycols (PEGs). Examples include 1 *M* NaCl, 1.25 *M* ammonium sulfate, 0.2 *M* CaCl_2_ and a large range of PEGs commonly used for crystallization (PEG 400–8000 at a concentration of up to 30%). The stability of the agarose-based stream is a function of its specific viscoelastic and surface-tension properties; notable variations were observed over the range of protein crystal compositions tested. The most stable stream was achieved with protein crystals that contained high-molecular-weight PEGs (>2000) as the precipitant. Furthermore, agarose is also compatible with organic precipitants such as 2-methyl-2,4-pentanediol (MPD). Dissolving the agarose in different precipitants in some cases decreased the viscosity. In these cases, the agarose concentration was adjusted to 9%, which increased the viscosity and the ability to form a stable stream. An especially challenging case for crystal delivery in agarose was the large membrane protein complex PSI (a trimer of 1080 kDa containing 36 protein subunits and more than 300 noncovalently bound cofactors), which crystallizes at low ionic strength without the addition of any salt or precipitant [Jordan *et al.*, 2001 ▸; Hunter & Fromme, 2011 ▸; Chapman *et al.*, 2011 ▸; the final crystallization buffer contained only 5 m*M* 2-(*N*-morpholino)ethanesulfonic acid (MES) pH 6.4 and 0.02% β-dodecyl­maltoside (β-DDM) detergent]. PSI crystals dissolve in the presence of glycerol or salt and do not tolerate the addition of any organic solvents or PEGs. To prevent the freezing of the PSI crystals in the agarose stream in high vacuum, we stabilized the PSI crystals by embedding them in agarose prepared with the PSI crystallization buffer and with 0.6 *M* sucrose. After optimization of the crystallization buffer, PSI crystals could be embedded into agarose and delivered to the XFEL beam in agarose with suitable crystal delivery stream stability using the crystallization buffer with sucrose described above in 9% agarose.

The agarose stream was tested using the Coherent X-ray Imaging (CXI) instrument at the Linac Coherent Light Source (LCLS) at the SLAC National Accelerator Laboratory in the vacuum-chamber setup (Boutet *et al.*, 2012 ▸) used for most of the SFX experiments published to date. Most of the SFX data were collected in the vacuum chamber, except for PSI, where the data shown in Supplementary Fig. S4 were collected in a new ambient-pressure setup with a helium environment. The advantage of using an ambient-pressure setup is that freezing by evaporative cooling is avoided. At ambient pressure cryoprotectant is not essential as dehydration occurs much more slowly than in vacuum.

The agarose stream relies on a high-velocity inert-gas (nitrogen) sheath to center and stabilize the emerging crystal jet extrusion (Weierstall *et al.*, 2014 ▸). This stability is required in order to reliably align the agarose stream with the XFEL beam axis. We observed a higher stability of the stream in vacuum compared with the ambient-pressure setup in the helium atmosphere. The lower stability of the stream at atmospheric pressure is presumably owing to a turbulent boundary layer at the interface between the inert-gas sheath stream and the surrounding ambient-pressure inert gas. Furthermore, the background is higher in the He atmosphere compared with the vacuum setup. For these reasons, vacuum operation of the stream is preferred when freezing can be avoided by the addition of PEG, glycerol or other cryo­protectants. SFX data for PC and PSII were collected using the vacuum-chamber setup and SFX data for PSI and PSII were collected using the helium ambient-pressure setup as described in §[Sec sec2]2.

**Figure 3:**
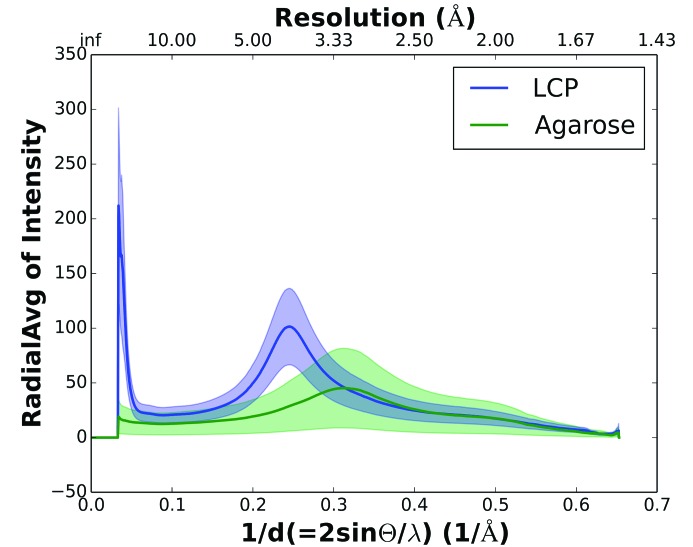
Diffuse background scattering comparison between agarose and LCP. 1/*d* (*x* axis) is plotted against the mean radial intensity over the total number of frames used from each medium (*y* axis). The blue line represents the mean radial intensity for LCP medium as a function of 1/*d* (or resolution in Å on the second *x* axis). The green line represents the mean radial intensity for agarose as a function of 1/*d*. The error or fluctuation in the radial intensity is quantified using the mean absolute deviation for both media, which is shown as a transparent gray region.

Fig. 3 ▸ shows a comparison of the X-ray scattering from the agarose stream compared with the LCP stream. We calculated the average scattered intensity from each medium delivered in a stream of the same width (50 µm) using detector-readout events that contained no crystal diffraction. We analysed 13 902 frames from the agarose stream data and 14 592 frames from the LCP stream data. Frames that contained no scattering from the jets/streams (owing to the jet/stream temporarily fluctuating out of the path of the X-rays) were easily recognized on the basis of their very low photon counts (∼10–20 detector units) and were excluded from the mean background calculation, leaving 9147 and 8326 frames with scattering from the LCP and agarose jets, respectively. Thereby, bias from large jet/stream flow instabilities was avoided in the calculation of the mean radial intensities for each medium. To reduce the influence of shot-to-shot variations in the XFEL pulse intensities, each frame was scaled to the readings from the gas-ionization detector upstream of the vacuum chamber at the CXI. Finally, the mean radial intensities from the LCP and agarose jets were scaled to be equal at a resolution of 2 Å, where neither medium should produce a background signal. As shown in Fig. 3 ▸, a broad peak corresponding to diffuse scattering from the lipid chains of LCP can be seen at 4.5 Å resolution. Diffuse scattering from agarose can be seen in the 3.3 Å region. The gray regions represent the mean absolute deviation around the mean. Overall, the background scattering from the agarose medium is roughly 2.3 times less than that from LCP in the diffuse-ring regions. Furthermore, LCP scatters strongly at very low resolution (>30 Å), while the low-angle scattering is very low in the agarose medium owing to the lack of long-range order and thus is ideal for large unit cells (Lawrence *et al.*, 2015 ▸). Indeed, LCP (Weierstall *et al.*, 2014 ▸; Liu *et al.*, 2013 ▸), mineral oil-based grease (Sugahara *et al.*, 2015 ▸) and petroleum jelly (Botha *et al.*, 2015 ▸) all result in higher background scattering than agarose, especially at low resolution (below ∼30 Å). Each of the media also produces diffuse scattering and/or Debye–Scherrer rings at 4–5 Å for LCP, 4–5 and 14 Å for mineral oil-based grease and 4.2 and 3.77 Å for petroleum jelly. The low background of agarose is not surprising considering that it is composed of 93% water and only 7% agarose, compared with 50% water and 50% lipids in LCP and 100% oil in grease or petroleum jelly.

The time available for data collection was limited to 4 h of protein crystal screening beamtime at LCLS. A full data set for PC crystals was collected using the vacuum setup at CXI (Figs. 3 ▸ and 4 ▸) during this time as well as a brief test run on PSII crystals. In addition, data were collected from PSII and PSI during short test runs using the ambient-pressure setup (Figs. 3 ▸ and 4 ▸). These complexes were chosen to demonstrate that agarose is an excellent carrier medium that can be used for SFX data collection from even the largest protein complexes containing noncovalently bound cofactors (Supplementary Figs. S3 and S4).

**Figure 5:**
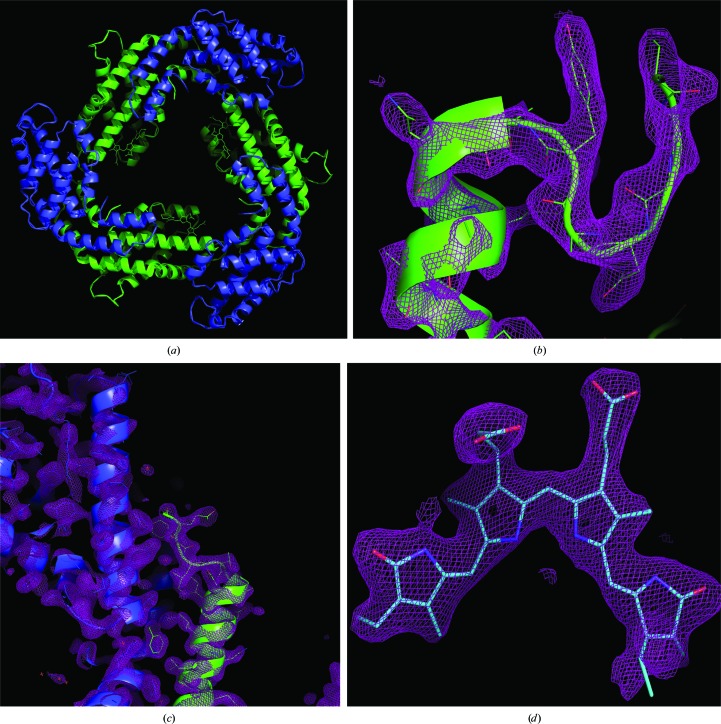
2*F*_o_ − *F*_c_ electron-density maps of PC. (*a*) PC trimer composed of two subunits, α (blue) and β (green), (*b*) an α-helix and loop from the α subunit contoured at 2.0σ, (*c*) α-helices from both subunits at 1.5σ and (*d*) the chromophore of PC at 1.5σ

The statistics of the PC data set are shown in Table 1 ▸. In 72 min of data collection, we collected 41 100 crystal hits from PC, of which 14 143 could be indexed in a hexagonal lattice with unit-cell parameters *a* = *b* = 153.4, *c* = 39.6 Å. A high multiplicity is essential for the determination of accurate structure factors by Monte Carlo integration (Kirian *et al.*, 2010 ▸) to average out the fluctuating parameters such as pulse intensity, partiality of reflections and crystal size distribution. The PC data set showed a high overall multiplicity of 250.6 and a multiplicity of 12.5 in the highest resolution shell (2.63–2.5 Å). The structure was solved using molecular replacement and the structure was refined with final values of *R*_work_ = 18.7% and *R*_free_ = 25.2% (see §[Sec sec2]2). Fig. 5 ▸ shows the electron-density map for the loops and side chains from subunit α (cyan) and subunit β (green), as well as a detailed view of phycocyanobilin, the chromophore of PC.

## 4. Conclusion   

In comparison to the commonly used GDVN liquid jet, which consumes protein crystal suspension at 10–25 µl min^−1^, the agarose delivery method presented here delivers protein crystals at a flow rate of only 160 nl min^−1^, reducing net protein consumption by two orders of magnitude. We have shown that the agarose medium might be suitable as a general delivery system for SFX of both soluble and membrane protein crystals and that it is compatible with a wide range of crystallization conditions as well as temperatures. The agarose jet can be used both in vacuum and at ambient pressure; so far, the stream has displayed better stability in vacuum. The agarose medium features lower X-ray scattering background compared with LCP or other viscous crystal delivery media such as mineral oil-based grease and petroleum jelly, especially at low resolution. Thus, agarose is an ideal SFX crystal delivery medium for protein crystals with large unit cells and medium-to-low resolution limits. The agarose delivery system is a low-cost, readily available medium for sample delivery of crystals of soluble and membrane protein complexes and is compatible with most commonly used precipitants, including various PEGs as well as high-salt conditions. Here, we have demonstrated that crystals can be embedded into the agarose medium post-crystallization. Furthermore, low sample consumption extends the SFX method towards protein complexes that are difficult to express and isolate in large amounts. Thus, this technique will allow structures of scarce proteins and systems that are difficult to crystallize in large quantities to be investigated by serial femtosecond crystallo­graphy.

## Supplementary Material

PDB reference: phycocyanin, 4z8k

Supporting Information.. DOI: 10.1107/S2052252515009811/mf5010sup1.pdf

## Figures and Tables

**Figure 4:**
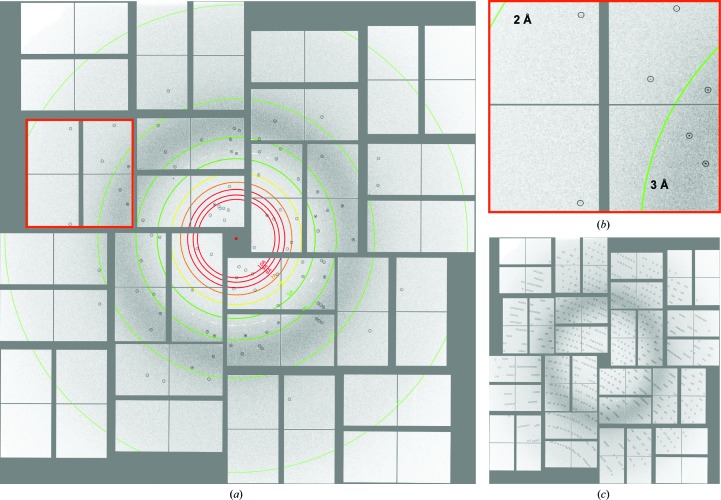
Single diffraction pattern of PC in agarose measured using the CXI at LCLS, with the red box magnified in (*b*) and predicted peak positions circled after indexing with *CrystFEL* (*c*).

**Table 1:** Phycocyanin data statistics Values in parentheses are for the highest shell.

Wavelength ()	1.33
Space group	*P*6_3_
Resolution ()	29.52.5 (2.552.50)
Unit-cell parameters (, )	*a* = *b* = 153.4, *c* = 39.6, = = 90, = 120
No. of crystal hits	41100
No. of indexed patterns	14143
Duration of data collection (min)	72
Unique reflections	18908
Reflections used in refinement	18871
*I*/(*I*)	3.2 (0.83)
Multiplicity	250.67 (12.5)
CC*	0.971 (0.487)
*R*_work_/*R*_free_ (%)	18.7 (32.7)/25.5 (35.5)
Completeness (%)	99.82
Average *B* factor (^2^)	38.34
