# Supplementary material for: A novel inert crystal delivery medium for serial femtosecond crystallography
Source: IUCrJ. 2015 Jun 30;2(Pt 4):421–30. doi: 10.1107/S2052252515009811 (PMC4491314; doi:10.1107/S2052252515009811)
Supplement: Supplementary file 1 [file m-02-00421-sup1.pdf]

IUCrJ

Volume 2 (2015)

Supporting information for article:

A novel inert crystal delivery medium for serial femtosecond crystallography

Chelsie E. Conrad, Shibom Basu, Daniel James, Dingjie Wang, Alexander Schaffer, Shatabdi Roy-Chowdhury, Nadia A. Zatsepin, Andrew Aquila, Jesse Coe, Cornelius Gati, Mark S. Hunter, Jason E. Koglin, Christopher Kupitz, Garrett Nelson, Ganesh Subramanian, Thomas A. White, Yun Zhao, James Zook, Sébastien Boutet, Vadim Cherezov, John C. H. Spence, Raimund Fromme, Uwe Weierstall and Petra Fromme

Table S1 Data quality statistics for phycocyanin.

Resolution (Å)	#Reflections	#Measurement	Multiplicity	Completeness (%)	CC*	SNR* (CrystFEL)
11.04	1013	1154949	1140.1	100.00	0.989	7.90
5.99	980	636800	649.8	100.00	0.981	5.67
5.02	959	558792	582.7	100.00	0.983	6.00
4.48	950	517920	545.2	100.00	0.983	6.37
4.11	939	422140	449.6	100.00	0.978	5.87
3.85	951	322650	339.3	100.00	0.976	5.01
3.64	952	264625	278.0	100.00	0.969	4.36
3.47	931	211411	227.1	100.00	0.959	3.81
3.33	951	145805	153.3	100.00	0.956	2.88
3.20	940	100473	106.9	100.00	0.907	2.45
3.10	925	85798	92.8	100.00	0.876	2.11
3.01	970	77473	79.9	100.00	0.894	1.86
2.92	911	59827	65.7	100.00	0.866	1.55
2.85	933	48409	51.9	100.00	0.802	1.43
2.78	965	39337	40.8	100.00	0.802	1.26
2.72	898	27798	31.0	100.00	0.737	1.12
2.67	924	21450	23.2	100.00	0.603	0.99
2.61	942	17822	18.9	100.00	0.574	0.82
2.57	947	14732	15.6	100.00	0.640	0.80
2.52	927	11574	12.5	100.00	0.487	0.83
**Overall**	**18,908**	**473,9785**	**250.67**	**100.0**	**0.971**	**3.19**

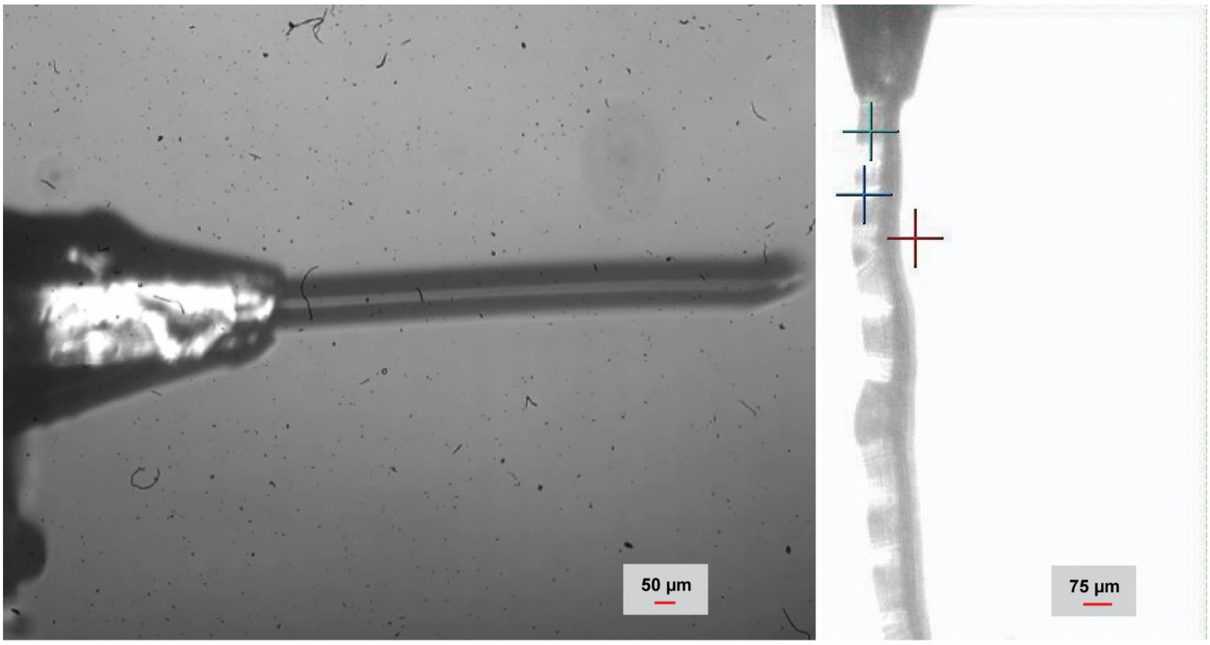

(*a*) (*b*)

Figure S1 Agarose stream extrusion. (a) Agarose stream with no embedded crystals extruded in vacuum in a test chamber (b) agarose stream with embedded crystals extruded in vacuum in the CXI chamber.

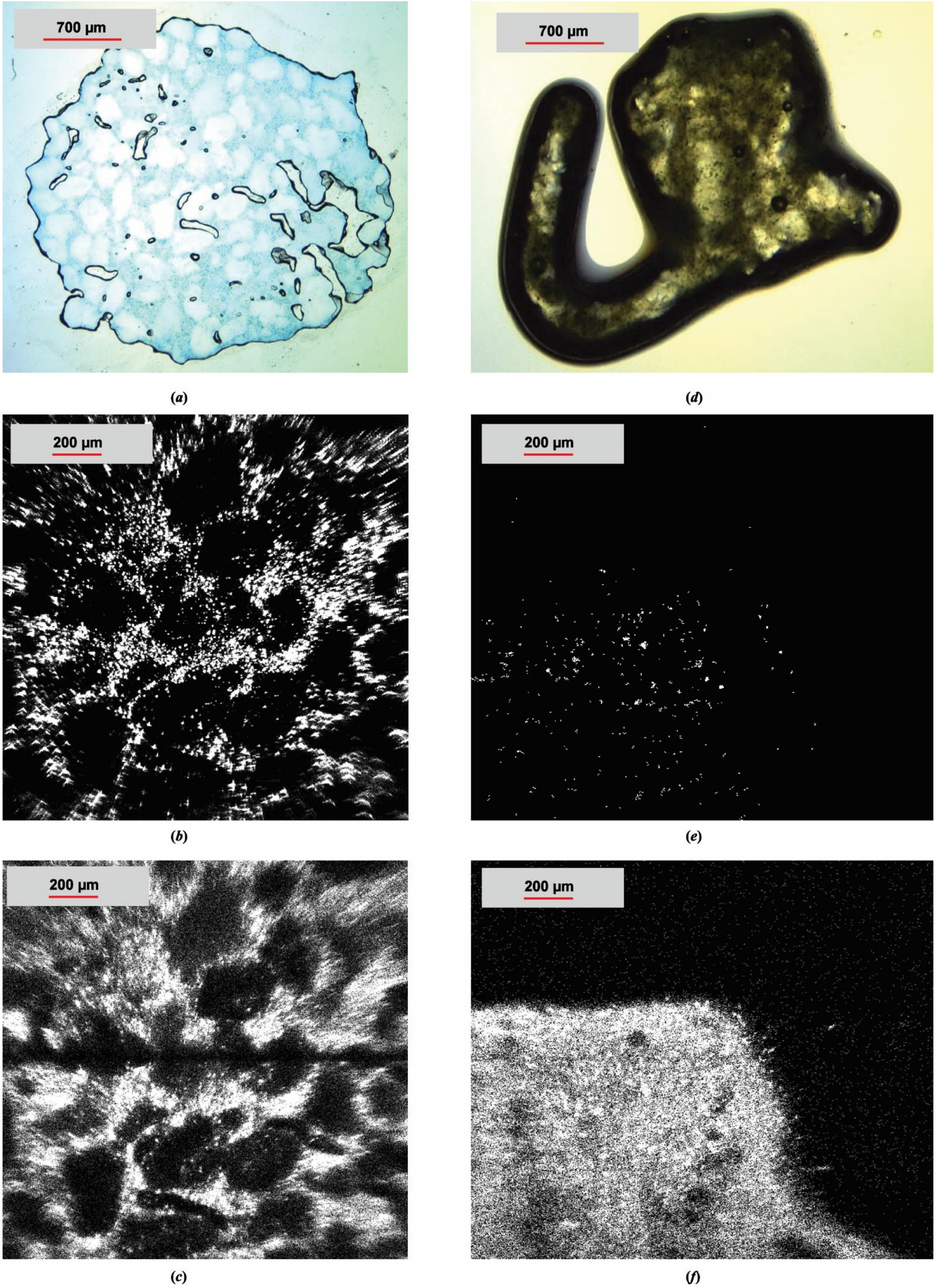

Figure S2 Characterization of PC and PSII embedded in agarose. (a) Brightfield of PC (b) second order non-linear imaging of chiral crystals of PC (c) ultraviolet two-photon excited fluorescence of PC microcrystals (d) brightfield of PSII (e) second order of non-linear imaging of chiral crystals of PSII (f) ultraviolet two-photon excited fluorescence of PSII.

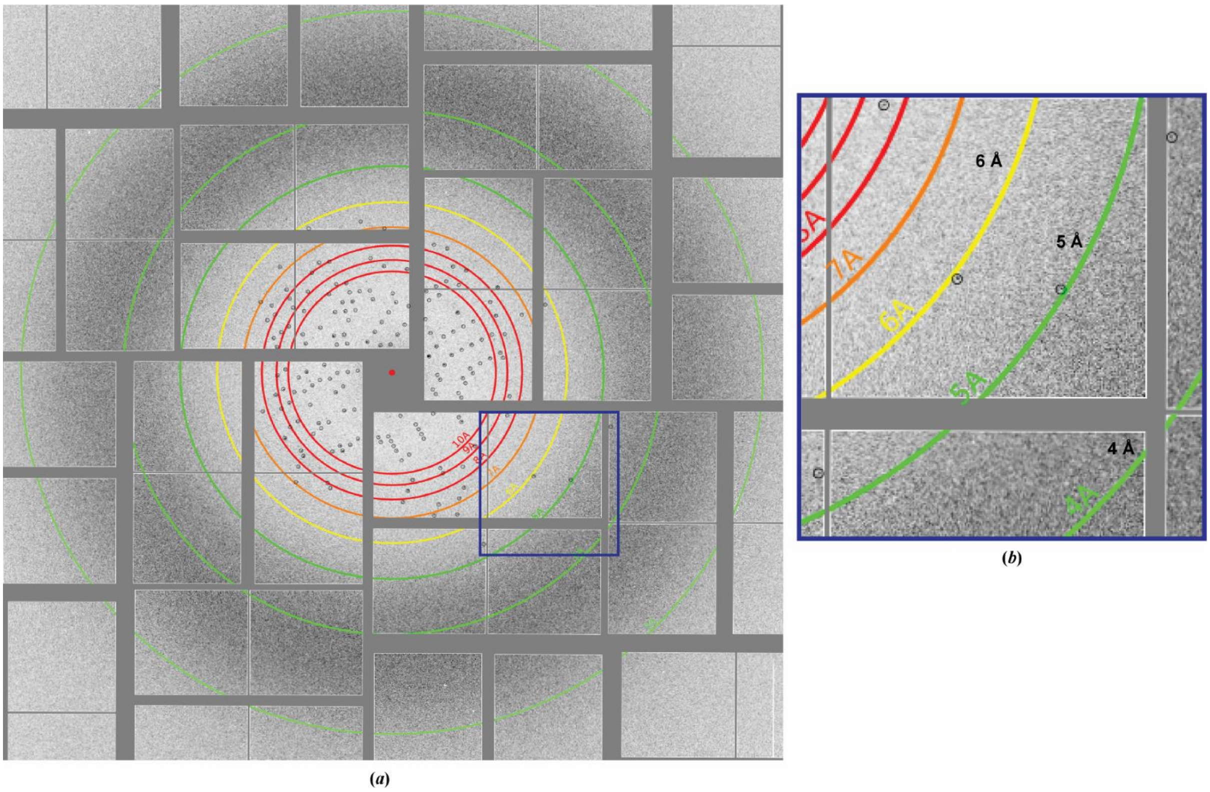

Figure S3 PSII embedded in agarose and delivered in a vacuum chamber. (a) Diffraction pattern from PSII in agarose, (b) magnified subset from (a).

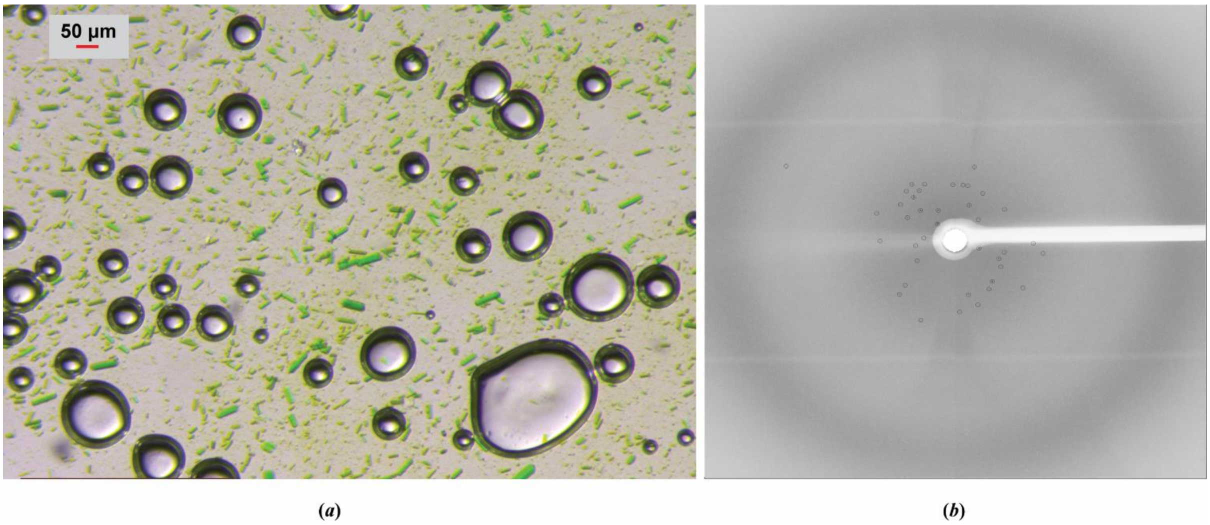

Figure S4 PSI embedded in agarose and delivered in a helium atmosphere. (a) Rod-like PSI crystals embedded in agarose, (b) diffraction pattern from PSI microcrystal in agarose.
